# Comprehensive Assessment of Degradation Behavior of Simvastatin by UHPLC/MS Method, Employing Experimental Design Methodology

**DOI:** 10.1155/2018/7170539

**Published:** 2018-08-08

**Authors:** Maja Hadzieva Gigovska, Ana Petkovska, Jelena Acevska, Natalija Nakov, Packa Antovska, Sonja Ugarkovic, Aneta Dimitrovska

**Affiliations:** ^1^Research & Development, Alkaloid AD, Blvd. Aleksandar Makedonski 12, 1000 Skopje, Macedonia; ^2^Faculty of Pharmacy, University “Ss Cyril and Methodius”, Mother Theresa 47, 1000 Skopje, Macedonia

## Abstract

This manuscript describes comprehensive approach for assessment of degradation behavior of simvastatin employing experimental design methodology as scientific multifactorial strategy. Experimental design methodology was used for sample preparation and UHPLC method development and optimization. Simvastatin was subjected to stress conditions of oxidative, acid, base, hydrolytic, thermal, and photolytic degradation. Using 2^n^ full factorial design degradation conditions were optimized to obtain targeted level of degradation. Screening for optimal chromatographic condition was made by Plackett–Burman design and optimization chromatographic experiments were conducted according to Box-Behnken design. Successful separation of simvastatin from the impurities and degradation products was achieved on Poroshell 120 EC C18 50 × 3.0 mm 2.7 *μ*m, using solutions of 20 mM ammonium formate pH 4.0 and acetonitrile as the mobile phase in gradient mode. The proposed method was validated according to International Conference on Harmonization (ICH) guidelines. Validation results have shown that the proposed method is selective, linear, sensitive, accurate, and robust and it is suitable for quantitative determination of simvastatin and its impurities. Afterwards, the degradation products were confirmed by a direct hyphenation of liquid chromatograph to ion-trap mass spectrometer with heated electrospray ionization interface. This study highlights the multiple benefits of implementing experimental design, which provides a better understanding of significant factors responsible for degradation and ensures successful way to achieve degradation and can replace the trial and error approach used in conventional forced degradation studies.

## 1. Introduction

To meet the demands of modern pharmaceutical analysis, the employment of chemometrical tools in every possible way during analysis is necessary, since many variables can be simultaneously controlled to achieve the desired results through limited experimental trials. The use of an experimental design (DoE) approach by which multivariate data can be handled and fitted to an empirical function is justifiable because it offers a better choice over one factor at time (OFAT) for identification and control of critical factors [1, 2].

In this direction, use of such systematic approach would be a necessity for any extensive study, such as forced degradation studies (FDS) for stability assessment of the active pharmaceutical ingredients (APIs) and finished dosage forms (FDFs). However, despite availability of diverse literature reports that defines the concept of forced degradation, detailed information about a forced degradation strategy is not provided and the experimental conditions to conduct forced degradation are described in a general way without description of the exact stress conditions to be applied [3–10]. Generally, a trial and error approach is adopted to select the strength, temperature, and time of exposure to achieve loss of active substance from 5 to 20% [5, 11, 12]. Till date, far from our knowledge, none of the reported analytical procedures describe simple and satisfactory sample preparation methodology where the influences of the stressor strength, time of expose, and temperature are evaluated in detail. Due to the considerable cost, time consumption, scientific expertise, and high incidence of random results, the need of more systematic approach is recognized.

The objective of this study was to present optimization of forced degradation condition using DoE.

Simvastatin (SIM) was chosen as model API for couple of reasons: (1) because of the great scientific community interest for its potential use in brain diseases and different types of cancers besides its well-known antihyperlipidemic activity and (2) due to its proven instability [13].

This study focuses on systematic evaluation of SIM instability in hydrolytic, oxidative, or photolytic condition, as currently available data mainly indicate the instability of simvastatin as a part of selectivity of the stability-indicating methods, while the influences of the stressor straight, time of expose, and temperature were not evaluated in detail [14–16].

Therefore, the goals of the present study are to explore the degradation behavior of SIM under different stress conditions (acidic, alkaline, oxidative, thermal, and photolytic) using simplified FDS by adopting DoE concept.

The research aimed to employ DoE approach for development and optimization of UHPLC/MS method to resolve all the possible degradation products, followed by method validation studies for ensuring the robust performance.

The proposed methodology would enable studying the combination of conditions where optimal degradation is obtained, as well as evaluation of the effect of each factor with the change in level of the other factors. This approach was chosen because it is efficient and easily accomplishable and allows interactions to be detected. Furthermore, this approach reduces the number of experiments, time, and cost and obtains good prediction of desired degradation.

## 2. Materials and Methods

### 2.1. Chemicals and Standards

Simvastatin CRS (purity 99.7% “as is”), Simvastatin impurity E (Lovastatin), and Simvastatin for peak identification were provided by European Directorate for the Quality of Medicines and Health Care Council of Europe (EDQM-Strasbourg, France). Simvastatin impurity B (Simvastatin acetate ester), Simvastatin impurity C (Anhydro simvastatin), and Simvastatin impurity D (Simvastatin dimer) were purchased by LGC GmbH, Im Biotechnologiepark, TGZ II, Germany. Also, in house standard of methyl simvastatin was used.

Simvastatin API samples with certificate of suitability to the monographs of the European Pharmacopeia (CEP) were kindly supplied by Teva Pharmaceutical Industries Ltd., Israel.

All the reagents used (acetonitrile, ammonium formate, formic acid, sodium hydroxide, hydrochloric acid, and hydrogen peroxide) were analytical grade, purchased from Merck (Darmstadt, Germany). Water was purified by a Werner water purification system.

Regenerated cellulose membrane syringe filters with pore size 0.2 *μ*m, purchased from Phenomenex (Torrance, CA. USA), were used.

### 2.2. Experimental Conditions

#### 2.2.1. Ultra High Performance Liquid Chromatography (UHPLC)

Chromatographic analysis was performed on Waters Acquity Ultra Performance (Waters Corporation, Milford, USA) equipped with a Binary Solvent Manager, UHPLC Column Compartment, Sample Manager Heater/Cooler and autosampler, and photo-diode array detector. Instrument control, data acquisition, and processing were done by using Empower 2 build 2154 software.

The separation was performed on Poroshell 120 EC C18 50 × 3.0 mm, 2.7 *μ*m (Agilent Technologies, USA), using buffer (20 mM ammonium formate, pH 4.0) and acetonitrile (ACN) as a mobile phase in a gradient mode as follows: T (min)/ACN (%) 0/40; 10/40; 20/85; 25/85; 30/40; 35/40. The column temperature was 35°C. Flow rate was 0.7 mL/min. Injection volume was 10 *μ*L. The UV detection was performed at 248 nm.

#### 2.2.2. Liquid Chromatography-Tandem Mass Spectrometry (LC-MS)

The LC/MS analyses were conducted on Dionex UltiMate™ 3000 UHPLC-UV-DAD (Thermo Fisher Scientific, Waltham, MA, USA), interfaced with linear ion-trap mass spectrometer (LTQ XL) equipped with heated electrospray-ionization source operated in the positive ionization mode. Instrument control and results processing was done using Dionex Chromeleon 7.2 (for UHPLC-DAD analyses) and Thermo Xcalibur v2.2 SP1 (for UHPLC-DAD/MS analyses). Structural confirmation and fragment elucidation were performed using Mass Frontier v7.0.

Optimized mass parameters were as follows: ion source heater temperature was set at 280°C and capillary temperature at 200°C; capillary voltage was 20 V with collision energy 35 eV.

Nitrogen was used as nebulizing gas at pressure of 50 psi and the flow was adjusted to 10 L/min. MS data were acquired in the positive ionization mode. The full scan covered the mass range at* m/z* 100-1200. Collision-induced fragmentation experiments were performed in the ion trap using helium as collision gas, with voltage ramping cycle from 0.3 up to 2 V. Maximum accumulation time of ion trap and the number of MS repetitions to obtain the MS average spectra were set at 500 ms and 3, respectively.

#### 2.2.3. Screening and Optimization of the UHPLC Method Using DoE

*(1) Application of Plackett–Burman Design for Screening Significant Parameters*. The Plackett–Burman design (PB design) was used to study the effects of nine independent factors, i.e., molarity of ammonium formate (*x*_1_), flow rate (*x*_2_), wavelength (*x*_3_), volume of injection (*x*_4_), detector acquisition rate (*x*_5_), column temperature (*x*_6_), percent of organic modifier in the initial mobile phase composition (*x*_7_), different column lots (*x*_8_), and injector temperature (*x*_9_).

The range and the levels of experimental investigated variables are presented in Table 1. For each of the 12 experiments 3 solutions (diluent, system suitability solution, and standard solution) were injected. The experiments were performed in randomized order to minimize the effects of uncontrolled variables that may influence the results. Three responses were measured for each experiment: resolution (*Rs*) between impurity E and F (*y*_1_),* Rs* between Impurity G and SIM (*y*_2_), and* Rs* between impurities B and C (*y*_3_).

*(2) Application of Box-Behnken Design for Optimization of the Chromatographic Conditions*. Next in this study, the important chromatographic factors selected based on the obtained results from PB design were optimized by a Box-Behnken (BB) design.

The independent variables were pH of ammonium formate (*x*_1_), flow rate (*x*_2_) contents of ACN in the initial mobile phase composition (*x*_3_). The low, centre, and high levels of each variable are given in Table 2. Three responses were measured for each experiment:* Rs* between impurity G and SIM (*y*_1_), tailing of SIM (*y*_2_), and* Rs* between impurities B and C (*y*_3_).

A total of 15 tests (including 3 replicates of the centre point) were carried out in random order, in accordance with the BB design (Table 2).

### 2.3. Standard and Sample Preparation

#### 2.3.1. Standard Preparation

Standard solution of SIM in final concentration of 1 *μ*g/mL was used for quantitative determination. Standard solutions of all impurities were prepared individually and in a mixture to final concentration of 4 *μ*g/mL each, using ACN and water in ratio 50 : 50 as solvent.

7.5 mg of “simvastatin for peak identification”, corresponding to a mixture of SIM spiked with its related impurities (A, B, C, D, E, and F), was dissolved in 5.0 mL solvent and used as system suitability solution.

#### 2.3.2. Sample Preparation

In all experiments the concentration of SIM in the sample solution was 1000 *μ*g/mL. Simvastatin was subjected to stress under acidic, alkaline, oxidative, thermal, and photolytic conditions.

In the preliminary experiments, SIM was subjected to 0.1M HCI for 3 hours and 0.1M NaOH for one hour at ambient temperature. The oxidation stress was done with 30% H_2_O_2_ solution for 3 h, at ambient temperature. For thermal degradation SIM was exposed at 60°C for 24 hours. Photo degradation study was performed by exposing the drug powder, spread as a thin film in a transparent quartz Petrii plates covered with a transparent quartz cover and exposed to direct sunlight for one and two days. Additionally, control study in dark was run simultaneously. All stress studies were performed in amber color glassware to protect the solutions from light degradation.

*(1) Sample Preparation according to Full Factorial Design for Acid, Alkali, Oxidative, and Thermal Degradation*. The forced degradation experiments set-up based on 2^n^ full factorial design was performed. The experiments were designed considering variables including time of exposure, temperature, and stressor strength at two levels.

Acid and alkali degradation was performed using 2^3^ factorial design (three variables considered at two levels: 0.01 M and 0.1 M HCl/ NaOH heated at 25°C and 40°C for 15 and 45 min). Set-up of eight experiments for each stressor was conducted as described in Table 3.

At the end of exposure, the samples were neutralized with appropriate amount of NaOH or HCl respectively (0.01 M or 0.1 M) and diluted to final concentration of 1000 *μ*g/mL with solvent.

For oxidative degradation also three variables were considered at two levels (the high level for H_2_O_2_, temperature and time of exposure were 30%, 40°C and 60 minutes, and the low levels were 3%, 25°C and 15 minutes, respectively).

2^2^ factorial designs were conducted to set up thermal degradation where the high-level values were 105°C and 5 h and the low levels were 80°C and 3 h, respectively.

### 2.4. Statistical Evaluation

The experimental design and statistical analysis of data for the optimization and robustness testing along with forced degradation sample preparation were performed with Design-Expert software, Version 7.0.0 (Stat-Ease, Inc., Minneapolis, MN, USA).

## 3. Results and Discussion

### 3.1. Optimization of Chromatographic Conditions

The method conditions were evaluated to obtain good quality of separation and ideal peak shape and maintain resolution between all impurities in minimum analysis time. Decisions concerning the type of stationary phase, solvent type, and water phase nature were made based on prior knowledge from literature. Although some methods have been developed for the determination of SIM and its impurities [14–18] including the two official methods reported in European Pharmacopeia and United State pharmacopoeia utilizing HPLC gradient elution, to the best of our knowledge, there are no references in the literature concerning chemometric approach to the development and validation of the UHPLC method, intended for the quantitative analysis of SIM and its impurities. These methods employed a time-consuming trial and error approach for giving potential information concerning the sensitivity of the factors on the separation and it did not provide the information concerning interaction between factors.

Therefore, within this study, science-based approach was employed to develop, optimize, and validate sensitive, robust, and cost-effective UHPLC method for determination of SIM and its impurities.

SIM and its impurities have very similar physical-chemical properties. The log*P* values are 4.39, 3.85, 4.94, 5.55, 3.68, 4.12, and 3.68 for SIM, impurity A, impurity B, impurity C and impurity E, impurity G, and impurity F, respectively (Table S1, supplementary material). The C_18_ packing columns were shown to be the most suitable according to the lipophilic nature of the compounds. Initially, four columns were examined (Zorbax Extend C_18_, Zorbax Eclipse C_18_, Zorbax XDB C_18_, and Poroshell 120 EC C18) and it was decided to continue the investigation on Poroshell 120 EC C18 50 × 3.0 mm, 2.7 *μ*m. This decision was based on the properties of this column, which is packed with specific, spherical core shell particles, allowing high efficiency of separation. The use of this column enabled tight, symmetrical peak with good resolution between impurities E and F. Among organic modifiers used in HPLC, it was decided to use ACN based on* Rs* between impurities E and F, critical* Rs* between B and C, and shorter analysis runtime. The addition of buffer was inevitable, and several buffers suitable for LC-MS analysis were examined (ammonium formate, ammonium acetate, and trifluoroacetic acid). Concentrations of these buffers were varied in the range from 5 to 20 mM (Table 1). Next, in order to obtain complete information about method behavior, nine factors were assessed in twelve experiments according to PB design (Table 1). PB design was chosen because of its high efficiency with respect to the number of runs required. The model was validated by the analysis of variance (ANOVA). The statistical analysis showed (data presented in Table S2, supplementary material) that the model represents the phenomenon quite well and the variation of the response was correctly, thus useful in predicting the effects of the factors on the selected responses.

**Figure 1:**
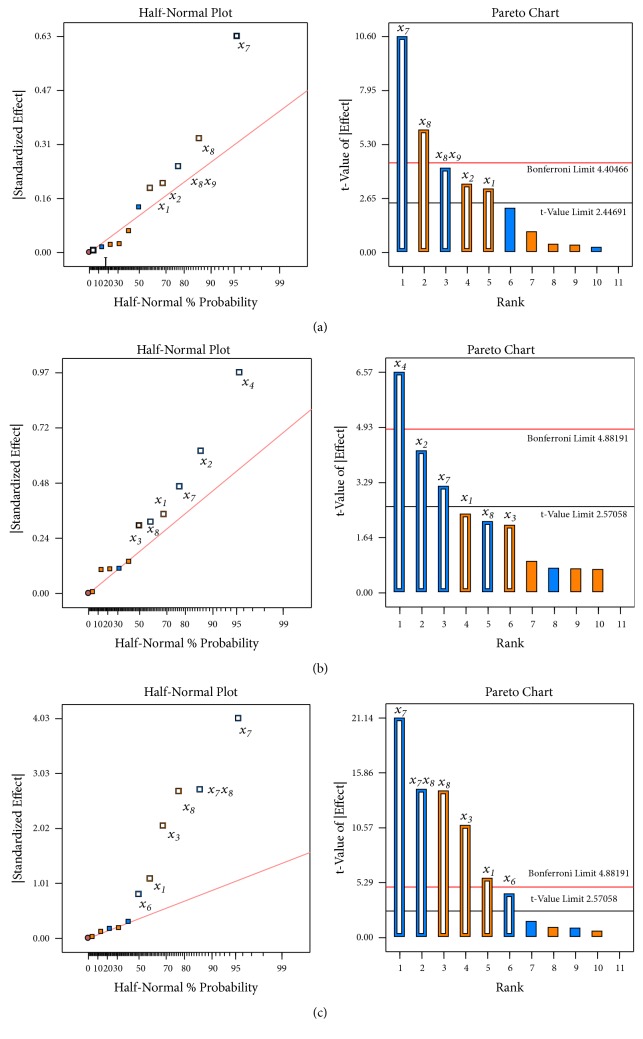
Half-normal plot and Pareto chart showing the significant effects based on the observation of Plackett–Burman design for the investigated responses (a)* Rs* between impurity E and F (*y*_1_); (b)* Rs* between SIM and impurity G (*y*_2_); (c)* Rs* between impurity B and C (*y*_3_) where *x*_1_ is molarity of ammonium formate (mM); *x*_2_ is flow rate (mL/min); *x*_3_ is wavelength (nm); *x*_4_ is volume of injection (*μ*L); *x*_5_ is detector acquisition rate (Hz); *x*_6_ is column temperature (°C); *x*_7_ is percent of organic modifier in the initial mobile phase composition (%); *x*_8_ is column of the same composition but of different lot; and *x*_9_ is injector temperature (°C). Positive effects are marked with white bar box with orange frame and orange bar box for significant and insignificant factor, respectable. Negative effects are marked with white bar box with blue frame and blue bar box for significant and insignificant, respectable.

Next, the qualitative contribution of each factor and, respectively, responses were analysed for all various conditions of degradation. Each response coefficient was studied for its statistical significance by half-normal plot and Pareto charts as shown in Figure 1. Pareto charts establish t value of the effect by two limit lines, namely, the Bonferroni limit line and t limit line. Coefficients with t value of effect above the Bonferroni line are designated as certainly significant. Coefficients with t value of effect between Bonferroni line and t limit line are termed as coefficients likely to be significant, while t value of effect below the t limit line is statistically insignificant.

Pareto chart analyses revealed that the molarity of ammonium formate, percent of organic modifier in the initial mobile phase composition (%), and column type have significantly affected all investigated responses.

Molarity of ammonium formate have been shown to have positive effect on all responses meaning that higher molarity revealed increased resolution and therefore all experiments in the optimization phase are performed using 20 mM ammonium formate. As a critical parameter pH value in range ±2 units were evaluated.

The statistical evaluation shows that the ratio of the mobile phase composition has a large effect on peak separation, which decreases with increasing organic portion. A negative relationship between flow rate varied from 0.3 to 0.7 mL/min and resolution between SIM and impurity G, and a positive relationship with resolution between impurities E and F was observed.

Additionally, an important factor affecting all responses was the column batch. It is very likely that during the ongoing investigation, column aging occurs and the related change in chromatographic separation might cause an inconclusive statistical evaluation. The other parameters investigated showed only little influence on resolution as displayed in Figure 1.

Hence, systematic scouting resulted in selection of three key critical parameters (flow rate, pH of buffer, and ACN content), which were optimized using BB experimental design (Table 2). Among the various experimental designs, BB design, as response surface design was preferred for the prediction of nonlinear response, due to its flexibility, in terms of experimental runs and information related to the factor's main and interaction effects. The model was validated by analysis of variance (ANOVA) using Design-Expert software (data presented in Table S3, supplementary material). Decision concerning the evaluated responses was made taking into consideration problems in the method described in European Pharmacopeia, where unsatisfactory separation between E/F and B/C is seen.

Comparison of different proposed models from experimental trials for all responses favored quadratic model as best fitted model. The data revealed in Table S3 indicates that the chosen quadratic models fit the data well and have high predictive powers for new observations. To get more realistic model insignificant terms with corresponding value higher than 0.05 were eliminated through backward elimination process. The coefficients of the second-order polynomial model were estimated by the least square regression analysis, and the function of responses related to the three selected factors was obtained. The obtained results are presented in Table S3.

From the obtained results it could be concluded that* Rs* between impurity G and SIM is negatively influenced by the content of ACN and positive influenced by the flow rate.

On the other hand,* Rs* between peak of impurities B and C is negatively influenced by pH of the water phase.

All the selected variables significantly influence peak symmetry, but the values obtained for the peak tailing ranged from 1.02 to 1.59 being therefore within the acceptable limits (≤2) for all determinations.

Also, the results suggested that two-factor interactions of investigated factors were significant as well, indicating the necessity of their simultaneous influence examination rather than isolated single factor at the time evaluation. Furthermore, the quadratic term indicates a nonlinear curvilinear trend.

**Figure 2:**
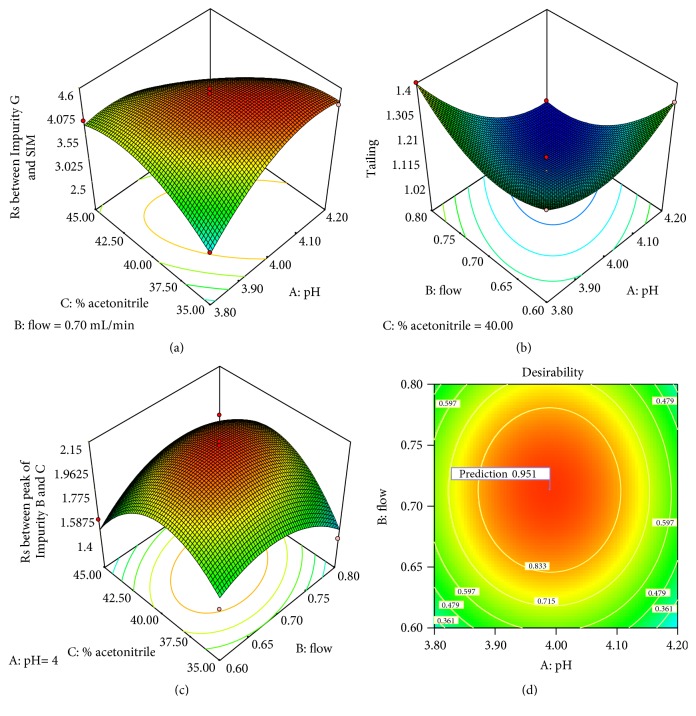
3D surface plot representing the (a)* Rs* between impurity G and SIM, (b) tailing factor, and (c)* Rs* between peaks of impurities B and C, as a function of % organic modifier, pH, and flow rate. (d) Desirability plot for optimization of the selected responses where the red area corresponds to the optimum chromatographic conditions while ACN content maintained constant at 40%. Color change from blue to red represents increasing response values (min
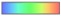
max).

In order to facilitate the visualization of factor interactions 3D response surface plots were created and presented in Figure 2. Response surface plots for all the evaluated responses were created keeping one factor constant (flow rate, content of acetonitrile and pH for* Rs* between impurity G and SIM; tailing and* Rs* between peak of impurities B and C as chosen responses, respectably)

Analyzing Figures 2(a) and 2(c), it can be concluded that simultaneous increase of pH of the water phase and decrease of ACN content in the mobile phase tend to increase* Rs* between impurity G and SIM, and simultaneous increase of ACN content in the mobile phase and flow rate enhances the* Rs* between peak of impurities B and C.

Direct determination of optimal factor setting was very difficult regarding the number and the antagonist influence of interaction and quadratic terms implicated in the model; therefore, optimal chromatographic conditions were chosen using desirability function, where* Rs* between peaks pair as critical parameter was considered at maximal.

No specific limitations were imposed to the tailing factor, as its value falls within the acceptable range in all cases in the experimental model (Figure 2(b)). From the desirability plot presented in Figure 2(d), it can be concluded that a set of coordinates producing high desirability value (D = 0.943) were pH value of 4, flow rate of 0.7 mL/min, and 40% ACN. These conditions were selected for further validation.

**Figure 3:**
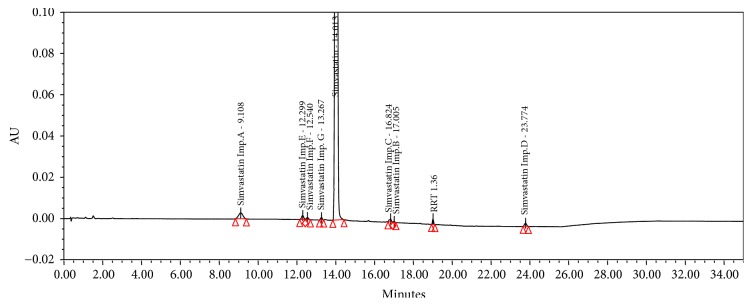
Representative chromatogram of peak identification solution.

The representative chromatogram of the peak identification obtained under optimized conditions is presented in Figure 3. Under the proposed chromatographic conditions, satisfactory separation (*Rs* 4.4) of SIM and impurity G was achieved, and the method can separate all known impurities with resolution more than 1.5, which is much better than obtained with existing monograph methods.

As it can be seen, the proposed methodology represents an efficient and easily accomplishable approach to resolving the problem of searching for optimum RP-UHPLC conditions.

Optimization of chromatographic method using experimental design methodology allow improvement of the accuracy and precision of the method by achieving better chromatographic separation of simvastatin from interfering chromatographic peak of impurity G. DoE approach is a systematic, scientifically approach that can reveal information that can be easily overseen when applying one factor at a time approach for method development and optimization. Additionally, it saves time investing the possible interaction between variables.

Employing such an approach, we have obtained the maximum amount of information with the smallest possible number of experiments. It provides an improved perspective and knowledge of the analytical procedure. The main advantage of this approach is evident: all the factors that potentially influence the separation can be studied simultaneously.

### 3.2. Method Validation

The developed and optimized method was validated as per ICH guidelines [19]. The validation results indicate than the method is specific, linear (0.4 to 6.0 *μ*g/mL for all impurities and 0.4 to 1.5 *μ*g/mL for SIM), accurate, and precise (Table 4).

Stability of the standard solution and sample solution was evaluated by analyzing the same sample immediately after preparation and after time interval (0, 18, and 38 h) by keeping the solution at room temperature. From the stability study, it was concluded that both standard and samples are stable for 38 hours in room temperature. The difference in % between centrifuged and filtered solutions was found to be within the limits (≤2) when samples are filtered through regenerate cellulose (RC-0.2 *μ*m) membrane filter, so this is suitable for filtration.

To test the capacity of this newly developed analytical procedure to withstand small deliberate changes in the method, various factors within the robustness testing were deliberately changed, like: column temperature (±5°C), organic content of mobile phase (±5%), flow rate (±0.1 units), and wavelength (±2 nm).

Robustness study confirmed that method could be considered robust because changes of factors in defined ranges do not influence the responses (Table 5).

### 3.3. LC-MS/MS Study on Forced Degradation Samples

The proposed method was transferred to a UHPLC/MS system to carry out deeper analysis of the behavior of SIM. Mass spectra and fragmentation patterns of all impurities were recorded, analyzed with Mass Frontier 7.0 fragmentation software, and confirmed by data published in literature [17, 18]. The obtained results are presented in Table S4, supplementary material. All the specified impurities [20], as well as unspecified impurity methyl simvastatin, were confirmed using commercially available standards. Additionally, other unknown impurities seen in a sample obtained by ‘worst-case forced degradation' samples were evaluated. Among these, the highest peak was unspecified impurity with relative retention time (RRT) 1.16 related to SIM retention time. As can be seen from the results presented in Table S4, the MS spectra of the impurity with RRT 1.16 revealed modifications of SIM molecule on the lactones ring. The finding that the molecular mass of this impurity has mass 46 amu higher compared to SIM implies that it is a derivative of SIM (presented in Table S1). The fragmentation patterns of this impurity follow distinct fragmentation as SIM, which are in good agreement with literature [18]. The proposed method was found suitable for detection of both specified and main unspecified degradation products in the samples obtained by force degradation.

### 3.4. Optimization of the Sample Preparation and Experimental Condition for Forced Degradation

#### 3.4.1. Stability of SIM under Different Forced Degradation Conditions

SIM according to the results obtained from our preliminary experiments, described in Section 2.3.2, was found to be susceptible to degradation under acid and alkali hydrolysis and it is slightly degraded under photo, oxidative, and thermal degradation. According to the requirements stated in European Pharmacopeia, two specified impurities (impurity E and impurity F) should be evaluated [20].

In the preliminary experiments, it was found that impurities E and F remained unaffected by all stress conditions applied. Simvastatin impurity B and impurity G were not detected at all, whereas impurity C only slightly increased in heated sample solution. Impurity D is proven to be formed in lactonization and was found in all stress conditions applied, with maximal obtained degradation of 0.08%. Simvastatin impurity A was found to be sensitive to all stress conditions applied, especially after acid and alkali hydrolysis. Therefore, beside the percentage of total impurities, amount of formed impurity A was chosen to be evaluated with the DoE approach, together with the major unspecified impurity with RRT 1.16.

#### 3.4.2. Optimization of Various Forced Degradation Conditions by DoE Approach

*(1) Selection of Independent Variables, Dependent Variables, and Model*. The* independent variables* evaluated by the full factorial DoE enclosed in Section 2.3.2. (1) were selected based on preliminary experiments and detailed literature survey which provided valuable information about the experimental region and definition of factors intervals. Stressor strength, time of exposure, and temperature were identified as factors which should be analysed.

Selection of the type and concentrations of stressor for oxidative degradation, acid, or base was made considering the results from our previous conducted preliminary experiments. HCl and NaOH at range of 0.01 M and 0.1 M were evaluated as suitable reagents for hydrolysis. H_2_O_2_ with concentration of 3% and 30% was used for oxidative forced degradation. The effect of temperature on acid, alkali, and oxidative degradation of SIM was studied at two levels 25°C and 40°C, respectively. Usually, hydrolytic degradation usually is performed at room temperature, and if no degradation is observed, then the temperature can be increased. However, implementing the DoE approach enables simultaneous evaluation of the effect of the temperature in just few experiments.

In order to gain information about degradation in short time, the time of exposure was chosen based on the minimum length. The low was set at 15 minutes and the high level was set at 45 minutes.

Following the general recommendation stated in ICH guideline [21], thermal degradation studies were performed at 80°C and it was considered as low level and 105°C was considered as high level. Time of exposure was chosen to be 3 and 5 hours.

The regulatory guidance does not specify the initial concentration of a compound for FDS [21] and several studies recommended range from 100 to 1000 *μ*g/mL [5, 11, 12]. In order to get even minor decomposition products in the range of detection, initial concentration of 1000 *μ*g/mL was chosen for this study.

Amount of total impurities (%), Simvastatin impurity A, and unknown impurity RRT 1.16 were chosen as* dependent variables.*

*(2) Statistical Verification of the Proposed Model*. The adequacy of the proposed design was statistically assessed by several statistical criteria, such as coefficient of determination (R^2^), adjusted R^2^, predicted R^2^, and adequate precision. As can be seen in Table 6 the calculated values of the R-squared (R^2^ > 0.9 in all cases) and adjusted R^2^ indicate that the model reasonably fits the experimental data.

The predicted R-square value was in acceptable concordance with the adjusted R-square value for all responses. The differences between the predicted R values and the adjusted R values are small, and thus, they are in reasonable agreement.

Adequate precision defined as a signal-to-noise ratio greater than 4 is desirable, and the obtained ratio for all the responses indicated an adequate signal (Table 6).

*(3) SIM Degradation Behavior Evaluated by the Proposed Model*. The significance of the effects of each variable was evaluated by ANOVA and the obtained results are presented in Table 6. Values of coefficients *b*_1_ for *y*_1_ and *y*_3_, and especially the values of coefficients *b*_3_ for all responses, demonstrate that SIM under acidic condition is most affected by strength of HCl and time of exposure. Values of the coefficients for the two-factor interaction, *b*_13_ for all investigated responses, confirmed the main effects of these factors. Both factors have positive sign, meaning that increase of the concentration of HCl and longer exposition is followed by an increase of the amount of total formed impurities, especially amount of impurity A.

As can be seen from the analysis of the percent of formed impurity A, it follows the same pattern as the total impurities discussed above.

The amount of formed impurity with RRT 1.16 follows different pattern, and in this case its formation is mainly affected by the temperature.

The assessment of the simultaneous influence of the time of exposure and strength of HCL on the formation of impurity with RRT 1.16 was based on interaction coefficient b_13_ given in Table 6. The absolute value of the coefficient characterizes the magnitude of the effect, whereas the sign of the coefficient shows whether the increase of the factor value increases (“+” sign) or decreases (“–” sign). Values of the coefficients for acid degradation for amount of formed impurity with RRT 1.16 showed that the interaction between time of exposure and strength of HCl has a significant effect on the degradation process. Individually, time of exposure and strength of HCL were not significant within the range evaluated, at the 95% confidence interval. This highlights the advantage of the proposed methodology, because this synergistic interaction might be overseen by traditional approach for conducting the degradation studies. Also, significant interaction between temperature and time of exposure was observed (Table 6). For alkali hydrolysis all the investigated factors have effect on the degradation of SIM. It was found that the degradation rate of SIM (and formation of impurity A) is strongly dependent on the time of exposition. Evaluating the obtained results can be seen that SIM is very sensitive in alkaline conditions and in some of the experiments degradation of more than 50% was observed. Performing the experiments using DoE allows an overview of the degradation behavior in wider region, so the risk of obtaining irrelevant results from secondary degradation is minimized, thus pointing out the additional advantage of the proposed design.

In addition, same as observed in acid degradation, this approach provides important information on interactions between time of exposure and strength of NaOH and their effect of the formation of impurity with RRT 1.16. Individually, these variables were not significant within the range evaluated, at the 95% confidence interval, but their synergistic effect on the degradation process favored formation of impurity with RRT 1.16.

These analyses indicated that, for oxidative degradation, temperature and the time of exposure were most significant factors for all the evaluated responses. Additionally, the strength of H_2_O_2_ was important factor for formation of impurity with RRT 1.16.

As it could be expected in thermal degradation, the temperature has biggest impact. This kind of effect was expected because it is known that kinetic constants have exponential dependency with reaction temperature (Arrhenius law) and this has also been reported by other authors [22].

**Figure 4:**
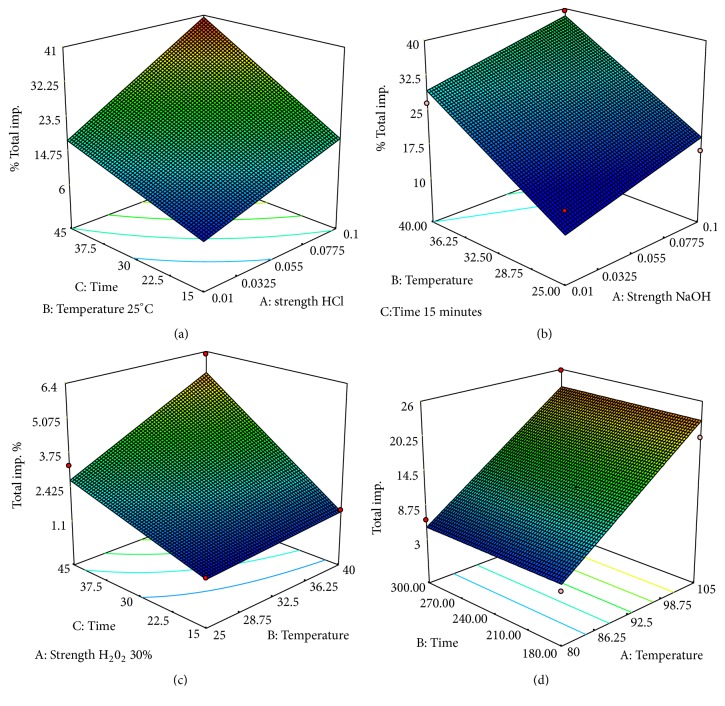
3D response surface plots showing the desired degradation under various conditions: (a) acid degradation; (b) alkali degradation; (c) oxidative degradation; and (d) thermal degradation. Color change from blue to red represents increasing degradation (min
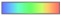
max).

*(4) Prediction Possibilities of the Proposed Model: Response Surface Methodology*. Next in the evaluation phase, response surface plots were generated for the most significant factors for each of the various degradation conditions, providing prediction of the conditions for optimum degradation (Figure 4). Each response surface plot represents a number of combinations of two test variables with all other variables at low levels.

The response surface plot for acid degradation was generated by keeping the temperature at minimum value 25°C (Figure 4(a)). As discussed before, increase of the concentration of HCl and longer exposition is followed by an increase of the amount of total impurities. In some of the experiments, the amount of total impurities (Table 4) was greater than 20%. Although there are references in the literature that mention wider recommended range, the more extreme conditions often provide data that are confounded with secondary degradation products; therefore, need for systematic approach for optimization is recognized [23–25].

The response surface plot for oxidative degradation was generated by keeping the H_2_O_2_ at maximal value (Figure 4(c)). This plot demonstrated that combination of temperature and time of exposure has positive effect meaning that longer exposure at temperature of 40°C will result with higher degradation, but maximum obtained degradation was about 7%. For oxidative degradation, it has been observed that 5% degradation would be achieved by treating with 30% H_2_O_2_ at 40°C for 45 min. When these conditions were adopted in practice, the resulting degradation was 5.68%.

The response surface plots obtained from thermal degradation study demonstrated that an increase in temperature from 80°C to 105°C favored the degradation significantly. The targeted drug degradation (Figure 4(d)) was obtained by heating the solution at 80°C for 5 h (5.15%), where minimum formation of impurity RRT 1.16 (0.05%) is achieved.

Photolytic studies were performed on classical manner and about 6.63% degradation has been obtained after exposure of UV light for 2 days.

**Figure 5:**
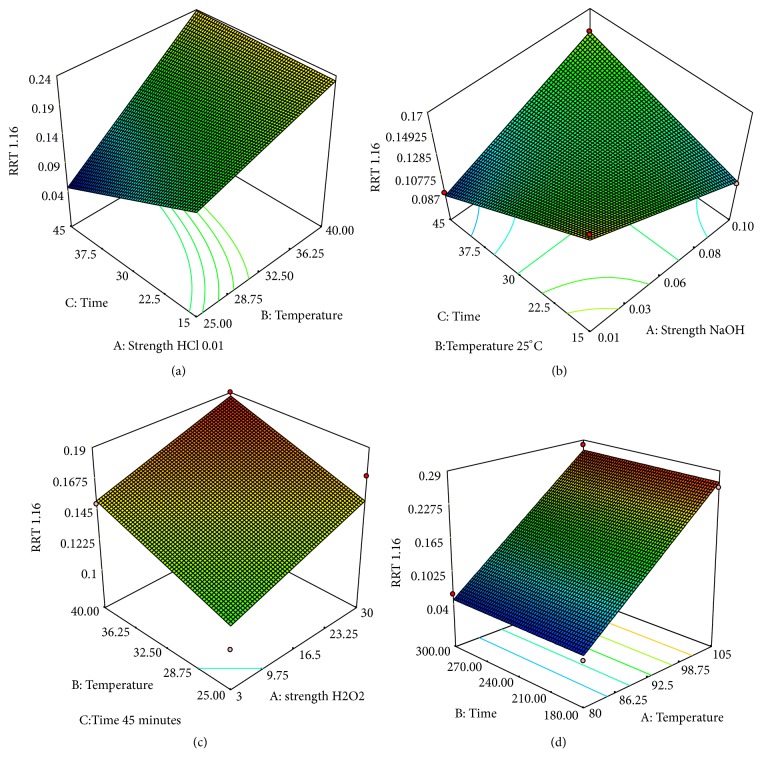
3D response surface plots showing the formation of impurity RRT 1.16 under various conditions: (a) acid degradation; (b) alkali degradation; (c) oxidative degradation; and (d) thermal degradation. Color change from blue to red represents increasing degradation (min
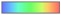
max).

Similarly, as for the total degradation product, response surface methodology can explain the formation of unspecified impurity with RRT 1.16. As can be seen in Table 3 and graphically presented in Figure 5, elevated temperatures favor formation of the impurity RRT 1.16. In acid degradation it was observed in maximal values, which is consistent with the evaluated literature data [18]. Some literature data suggests that formation of impurity with RRT 1.16 is not affected by applied stress condition (using 0.1 M HCl, 0.1 M NaOH, and 3% H_2_O_2_) [18]. However, the proposed experimental design indicated that with simultaneous evaluation of the time of the exposure and temperature it is possible to follow the degradation behavior of this impurity. Furthermore, it is possible to generate an explanatory model with possibility of relating these types of data in a single experiment.

*(5) Prediction Possibilities of the Proposed Model: Desirability Plot*. Optimal conditions were chosen using desirability function, where % of impurity (RRT 1.16) was targeted to 0.01 %, and targeted degradation was set in the range 5-20%, following the general literature recommendations [5, 11, 12].

No specific limitations were imposed to the % impurity A, as its value falls within the same range in all case in the experimental model as total impurities.

**Figure 6:**
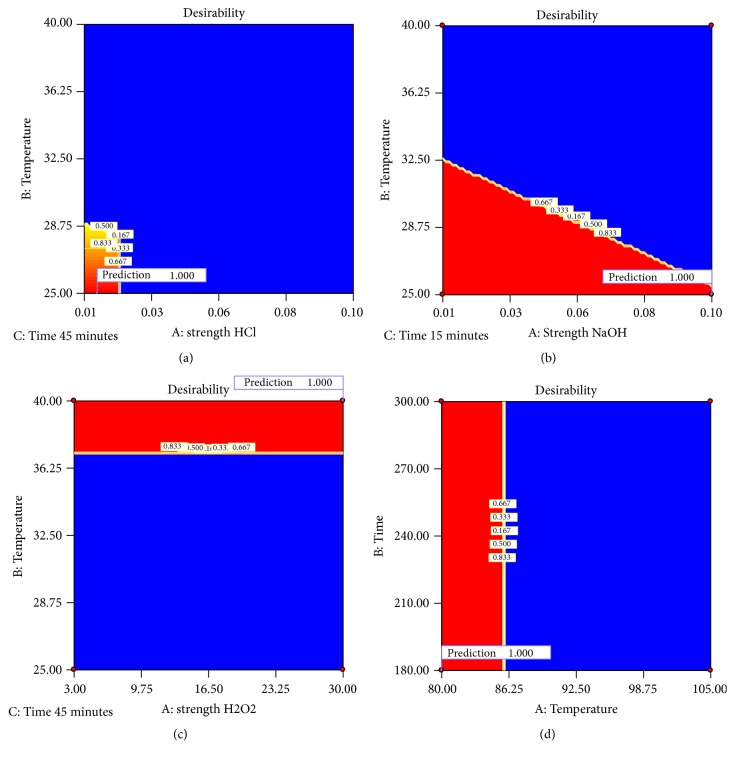
Optimization of the selected responses by means of the desirability function. The red area corresponds to the optimum conditions while time maintained constant: (a) acid degradation; (b) alkali degradation; (c) oxidative degradation; and (d) thermal degradation.

From the desirability plot presented in Figure 6(a), it can be concluded that a set of coordinates producing high desirability value (D = 1.00) are 0.01 M HCl, 25°C, and 43.5 minutes, which were selected as experimental condition in the verification study.

The predicted response values corresponding to the above optimum condition are given in Table 7. Comparison between obtained and predicted results was made and noticeable difference was not clearly observed (Table 7). The results of the experiments confirmed that the chosen model was adequate for reflecting the expected optimization. Good predictability of the desirability plot provides valuable information about proposed methodology, saving considerable amounts of chemicals and experimental time.

In alkali degradation response surface gradually increased with increasing the concentration from 0.01 M to 0.1 M NaOH and with increasing the temperature. However, as discussed above, this model could be used for prediction and optimal conditions were chosen using desirability function, where time of exposure was kept at minimal value.

From the desirability plot presented in Figure 6(b), it can be concluded that a set of coordinates producing high desirability value (D = 1.00) are 0.01 M NaOH at 25°C, followed by immediately neutralization with 0.01 M HCl.

#### 3.4.3. Advantages of the Proposed Model for Forced Degradation Studies

The proposed model is applicable for evaluation of degradation behavior of simvastatin. Generally, implementation of DoE in optimization of experimental conditions in forced degradation study give better data quality with less laboratory work and lead to a decrease in a cost of analysis. The use of DoE to identify theoretical values of variables for optimum degradation was successful, because when the proposed parameters were put in practice, the obtained results matched the predicted degradation.

In fact, the main significant advantage of the present methodology is the simplicity of the sample preparation since measurements are made directly on the liquid samples and optimum degradation was achieved in minimal experimental trials.

As explained with the degradation behavior of SIM through evaluation of total impurities and formation of unspecified impurity with RRT 1.16 under optimized degradation conditions, simultaneous evaluation of the time of the exposure and temperature gives information that can easily been overseen with traditional approach. The results enclosed in this study shows that it is possible to generate an explanatory model with possibility of relating these types of data in a single design methodology.

It is therefore hoped that the results reported here will provide useful guidelines for conducting forced degradation study.

## 4. Conclusions

The proposed methodology represents an efficient and easily accomplishable approach for searching optimum degradation conditions for conducting the stability studies on APIs. This study showed that DoE is an excellent tool and could successfully be used to develop empirical equation for the prediction and understanding of the degradation process. The obtained results showed sufficiently good correlation between the experimental data and predictive value throughout the studied parameters. This suggests that proposed full factorial design approach can replace the trial and error approach used to achieve optimum degradation in forced degradation studies.

The investigation also showed that chromatographic techniques coupled with chemometric tools provide useful information of separation and elution time, making this combined methodology a powerful analytical tool. The proposed optimized method for determination of simvastatin and its impurities gives rapid and efficient separation and represents an improvement over the existing reported methods especially in the terms of sensitivity and low cost per sample. The validation study supported the selection of the chromatographic conditions by confirming that the method was specific, accurate, linear, precise, and robust.

## Figures and Tables

**Table 1:** Plan of Plackett–Burman design and experimentally obtained results.

Number of experiments	Factor levels									Responses		
	*x*_1_	*x*_2_	*x*_3_	*x*_4_	*x*_5_	*x*_6_	*x*_7_	*x*_8_	*x*_9_	*y*_1_	*y*_2_	*y*_3_
1	20	0.7	228	7	25	35	60	lot 2	25	0.72	2.50	0.92
2	20	0.3	228	3	10	35	40	lot 2	5	0.92	4.25	1.25
3	20	0.3	228	7	10	45	40	lot 1	25	1.20	3.28	6.23
4	20	0.3	240	3	25	45	60	lot 1	5	1.02	3.72	1.22
5	20	0.7	240	7	10	35	60	lot 1	5	1.03	2.02	2.21
6	20	0.7	240	3	25	45	40	lot 1	25	1.56	3.44	7.78
7	5	0.7	228	7	25	45	40	lot 2	5	1.25	3.10	1.59
8	5	0.3	228	3	25	35	60	lot 1	25	0.88	3.72	2.16
9	5	0.7	228	3	10	45	60	lot 2	5	0.71	3.56	1.26
10	5	0.3	240	7	25	35	40	lot 2	5	1.08	3.89	6.41
11	5	0.7	240	3	10	35	40	lot 2	25	1.59	4.56	4.52
12	5	0.3	240	7	10	45	60	lot 1	25	0.80	3.35	2.53

*x*_1_: molarity of ammonium formate (mM); *x*_2_: flow rate (mL/min); *x*_3_: wavelength (nm); *x*_4_: volume of injection (*μ*L)*; x*_5_: detector acquisition rate (Hz); *x*_6_: column temperature (°C); *x*_7_: percent of organic modifier in the initial mobile phase composition (%); *x*_8_: column with the same composition but different lots (lot 1: USCFZ13194/B13243; lot 2: USCFZ13193/B13243); *x*_9_: injector temperature (°C);*y*_1_: Rs E/F; *y*_2_: Rs G/SIM; and *y*_3_: Rs B/C.

**Table 2:** Box-Behnken experimental design matrixes of the selected independent variables and studied responses.

Number of experiments	Factors levels			Responses		
	*x*_1_	*x*_2_	*x*_3_	*y*_1_	*y*_2_	*y*_3_
1	3.8	0.6	40	3.06	1.27	1.72
2	4.2	0.6	40	2.79	1.33	1.51
3	3.8	0.8	40	3.66	1.40	1.60
4	4.2	0.8	40	4.02	1.04	1.41
5	3.8	0.7	35	3.12	1.51	1.58
6	4.2	0.7	35	4.25	1.07	1.43
7	3.8	0.7	45	3.91	1.37	1.38
8	4.2	0.7	45	2.58	1.52	1.32
9	4.0	0.6	35	3.59	1.39	1.59
10	4.0	0.8	35	3.85	1.30	1.40
11	4.0	0.6	45	3.05	1.44	1.56
12	4.0	0.8	45	3.25	1.35	1.78
13	4.0	0.7	40	4.58	1.06	2.06
14	4.0	0.7	40	4.48	1.12	2.15
15	4.0	0.7	40	4.28	1.03	2.12

*x*_1_: pH of mobile phase; *x*_2_: flow rate (mL/min); *x*_3_: content of acetonitrile (%); *y*_1_: *Rs* between impurity G and SIM; *y*_2_: tailing factor SIM (T); and *y*_3_: *Rs* between impurities B and C.

**Table 3:** Experimental conditions and results from 2^n^ full factorial design for acid, alkali, oxidative, and thermal degradation.

	Experimental conditions			Acid degradation			Alkali degradation			Oxidative degradation			Experimental conditions		Thermal degradation		
Exp. No	Factor levels						Responses (%)						Factor levels		Responses (%)		
	*x*_1_	*x*_2_	*x*_3_	*y*_1_	*y*_2_	*y*_3_	*y*_1_	*y*_2_	*y*_3_	*y*_1_	*y*_2_	*y*_3_	*x*_4_	*x*_5_	*y*_1_	*y*_2_	*y*_3_
1.	-	-	-	7.32	0.15	6.83	15.28	0.17	11.23	1.09	0.03	0.12	-	-	3.98	0.04	3.38
2.	**+**	-	-	14.43	0.12	13.93	16.23	0.10	12.53	1.16	0.04	0.13	-	**+**	6.33	0.09	5.38
3.	-	**+**	-	5.79	0.25	5.04	26.65	0.12	15.23	1.45	0.06	0.14	**+**	-	20.12	0.26	17.10
4.	**+**	**+**	-	22.04	0.17	21.74	39.43	0.08	23.50	1.55	0.08	0.16	**+**	**+**	25.79	0.28	21.92
5.	-	-	**+**	16.71	0.05	16.39	37.25	0.09	35.89	2.19	0.10	2.04					
6.	**+**	-	**+**	41.05	0.09	40.73	48.32	0.15	40.23	3.29	0.17	2.16					
7.	-	**+**	**+**	18.76	0.23	18.47	58.23	0.11	45.62	4.79	0.15	2.56					
8.	**+**	**+**	**+**	39.89	0.29	38.32	68.89	0.19	58.26	6.28	0.19	2.89					

*x*_1_: stressor strength 0.01 M and 0.1 M HCl/NaOH or 3% and 30% H_2_O_2_ for hydrolysis and oxidative degradation respectable; *x*_2_: temperature 25°C and 40°C; *x*_3_: time 15 and 45 minutes; *x*_4_: temperature: 80°C and 105°C; *x*_5_: time 180 and 300 minutes; *y*_1_: amount of total impurities (%);*y*_2_: amount of impurity with RRT 1.16 (%); and *y*_3_: amount of impurity A (%). The high level of each factor was considered as “+" and low level as “−".

**Table 4:** Obtained results from validation of the proposed method.

	Imp.E	Imp.G	SIM	MSIM	Imp.B	Imp.C	Imp.D
System suitability							

Resolution	/	4.44	4.27	6.83	11.20	1.56	38.26
NTP	79895	110461	169356	268485	253612	184417	292527
T	1.01	1.02	1.04	1.05	1.07	1.02	1.02

Linearity ^(1)^							

Regression coefficient	0.9969	0.9994	0.9920	0.9980	0.9990	0.9970	0.9997
Slope	33806	15647	31722	10155	27192	21212	10765
Intercept	6077	388	6375	368	253	2171	401
Response factor	1.50	0.55	1.0	0.36	0.96	0.75	0.38

Precision ^(2)^							

Method precision	7.26	NA	NA	9.04	6.12	NA	NA
Intermediate precision (F –test)	1.86	1.02	NA	1.85	1.05	1.02	1.03

Accuracy given as recovery (%) ^(3)^							

50	98.8 ± 0.5	100.4 ± 0.6	100.5 ± 0.5	98.8 ± 0.6	99.5 ± 1.5	101.0 ± 0.2	100. 7 ± 1.0
100	98.3 ± 0.3	99.1 ± 1.4	100.2 ± 0.1	99.1 ± 0.7	99.7 ± 0.8	98.3 ± 0.9	100.3 ± 0.9
150	98.8 ± 0.2	99.1 ± 0.1	99.9 ± 0.6	99.1 ± 0.1	99.2 ± 0.2	101. 5 ± 0.2	99.6 ± 1.0

Sensitivity ^(4)^							

LOD (*μ*g/mL)	0.12	0.06	0.03	0.12	0.12	0.12	0.12
RSD	11.37	30.18	6.92	9.12	13.60	10.77	2.58

LOQ (*μ*g/mL)	0.4	0.4	0.1	0.4	0.4	0.4	0.4
RSD	3.98	1.30	9.29	4.79	1.58	3.30	6.64

(1) Nine solutions of SIM in the concentration ranging from 0.1 *μ*g/mL to 1.5 *μ*g/mL and nine solutions of all impurities in the concentration ranging from 0.4 *μ*g/mL to 6 *μ*g/mL were analyzed.

(2) The repeatability was shown by 6 replicate injections of the standard solution in concentration of 1 *μ*g/mL and the intermediate precision was performed on 6 samples in the two following days using the same equipment.

(3) Determined in triplicate at three concentration levels of 50%, 100%, and 150% by spiking the prequantified samples with a known amount standard of impurities.

(4) The LOD and LOQ were estimated at a signal-to-noise ratio of 3:1 and 10:1, respectively, for each impurity by injecting a series of dilute solutions with known concentration.

NA: not applicable, MSIM: methyl simvastatin, and RSD: relative standard deviation.

**Table 5:** Obtained results from validation of the proposed method**: **robustness.

	Simvastatin			Res E/F	Res G/SIM	Res B/C
	Rt (min)	T	NTP			
Flow (mL/min)						
0.6 mL/min	14.05	0.99	109188	1.52	4.48	1.66
0.8 mL/min	13.78	1.08	128239	1.50	4.46	1.53
Content of acetonitrile in initial phase						
35%	14.77	1.05	234052	1.53	4.05	1.61
45%	14.74	1.02	236896	1.58	4.17	1.66
Column temperature						
30°C	13.42	1.01	93614	1.51	4.68	1.89
40°C	13.83	1.02	109994	1.53	4.43	1.78
Another column	15.07	1.05	169635	1.59	4.47	2.15

**Table 6:** Statistical parameters of ANOVA and obtained regression coefficients for different degradation.

	R^2^	R^2^ Predicted	R^2^ Adjusted	Adequate precision	Regression coefficients							
					b_0_	b_1_	b_2_	b_3_	b_12_	b_23_	b_13_	b_123_
Acid degradation												

*y*_1_	0.9736	0.8943	0.9537	16.724	20.75	8.60	/	8.35	/	/	2.76	/
*y*_2_	0.9823	0.9293	0.9691	23.526	0.17	/	0.066	/	/	0.029	0.026	/
*y*_3_	0.9701	0.8806	0.9477	15.756	20.21	8.53	/	8.33	/	/	2.53	/

Alkali degradation												

*y*_1_	0.9810	0.9241	0.9668	22.878	38.79	4.43	9.51	14.39	/	/	/	/
*y*_2_	0.9590	0.8362	0.9283	15.000	0.13	/	/	8.7E^−3^		0.016	0.031	/
*y*_3_	0.9730	0.8920	0.9527	17.544	30.31	3.32	5.34	14.69	/	/	/	/

Oxidative degradation												

*y*_1_	0.9328	0.7312	0.8824	9.504	2.73	/	0.79	1.41	/	0.61	/	/
*y*_2_	0.9595	0.8382	0.9292	14.839	0.10	0.017	0.017	0.050	/	/	/	/
*y*_3_	0.9943	0.9771	0.9900	29.558	1.28	/	0.16	1.14	/	0.15	/	/

Thermal degradation												

*y*_1_	0.9439	0.7755	0.9158	8.203	14.05	8.90	/	/	/	/	/	/
*y*_2_	0.9918	0.9672	0.9877	22.000	0.16	0.11	/	/	/	/	/	/
*y*_3_	0.9383	0.7531	0.9074	7.797	12.06	7.45	/	/	/	/	/	/

Linear mathematical model of the measured response *y* = *b*_0_ + *b*_1_*x*_1_ + *b*_2_*x*_2_ + *b*_3_*x*_3_ + *b*_12_*x*_1_*x*_2_ + *b*_13_*x*_1_*x*_3_ + *b*_23_*x*_2_*x*_3_ + *b*_123_*x*_1_*x*_2_*x*_3_,

where **y** is the response [*y*_1_: amount of total impurities (%); *y*_2_: amount of impurity with RRT 1.16 (%); *y*_3_: amount of Simvastatin impurity A (%)], **x**_**i**_ is investigated factors [for acid, alkali, and oxidative degradation; *x*_1_ represents stressor strength (0.01 M and 0.1 M HCl/NaOH or 3% and 30% H_2_O_2_ for hydrolysis and oxidative degradation respectable); *x*_2_ represents temperature and *x*_3_ represents time of exposure. For thermal degradation *x*_1_ is temperature and *x*_2_ is time of exposure]; **b**_0_ is the intercept **b**_1_, **b**_2_ and **b**_3_, **b**_12_, **b**_23_, **b**_12_, and **b**_123_ as regression coefficients for the variables and interaction between the variables.

**Table 7:** Comparison of experimental and predictive values of different responses under optimal conditions.

Parameters	Predicted (%)		Obtained (%)		Predicted Error	
	Total imp.	RRT 1.16	Total imp.	RRT 1.16	Total imp.	RRT 1.16
Acid degradation	18.25	0.06	18.47	0.065	1.21	8.63
Alkali degradation	14.65	0.16	14.24	0.19	2.88	-15.78
Oxidative degradation	5.36	0.19	5.68	0.19	2.53	NA
Thermal degradation	5.18	0.05	4.88	BDL	-5.24	NA

Predicted error = (obtained values – predicted)/predicted *∗* 100 BDL (below disregard limit) (0.05%); NA: not applicable.

## Data Availability

The data supporting the findings of this study are available within the article and its supplementary materials. Raw data and additional information of this study are available from the corresponding author on request.

## Supplementary Materials

Supplementary Materials As supplementary materials Table S1, Table S2, Table S3, and Table S4 are included. Table S1: structure of the Simvastatin impurities. Table S2: statistical parameters of ANOVA and obtained results from Placket-Burman model. Table S3: the estimates of Box-Behnken regression analysis and statistical parameters of ANOVA. Table S4: Mass spectra fragments of SIM and its impurities.

## References

[1] Ganorkar S. B., Shirkhedkar A. A. (2017). Design of experiments in liquid chromatography (HPLC) analysis of pharmaceuticals: Analytics, applications, implications and future prospects. *Reviews in Analytical Chemistry*.

[2] Hibbert D. B. (2012). Experimental design in chromatography: a tutorial review. *Journal of Chromatography B*.

[3] Klick S., Muijselaar P. G., Waterval J. (2005). Toward a generic approach for: Stress testing of drug substances and drug products. *Pharmaceutical Technology*.

[4] Ngwa G. (2010). Forced degradation as an integral part of HPLC stability-indicating method development. *Drug Delivery Technology*.

[5] Blessy M., Patel R. D., Prajapati P. N., Agrawal Y. K. (2014). Development of forced degradation and stability indicating studies of drugs - A review. *Journal of Pharmaceutical Analysis*.

[6] Sharma M. K., Murugesan M. (2017). Forced Degradation Study an Essential Approach to Develop Stability Indicating Method. *Journal of Chromatography & Separation Techniques*.

[7] Alsante K. M., Martin L., Baertschi S. W. (2003). A stress testing benchmarking study. *Pharmaceutical Technology*.

[8] Bakshi M., Singh S. (2002). Development of validated stability-indicating assay methods—critical review. *Journal of Pharmaceutical and Biomedical Analysis*.

[9] Shubhangi S., Chaitali D., Joshi S. (2014). Force Degradation Study to Stability Indicating Method. *World Journal of Pharmacy and Pharmaceutical Science*.

[10] Aneesh T. P., Rajasekaran A. (2012). Forced Degradation Studies- a Tool for Determination of Stability in Pharmaceutical Dosage Forms. *International Journal of Biological & Pharmaceutical Research*.

[11] Ranjit S., Rehman Z. (2012). Current Trends in Forced Degradation Study for Pharmaceutical Product Development. *J Pharm Educ Res*.

[12] James S., Carolina N., Augsburger L. L., Brittain H. G., Hickey A. J., Hill C. (2005). Pharmaceutical Stress Testing © 2005. *Drugs and the Pharmaceutical Sciences*.

[13] Bifulco M., Endo A. (2014). Statin: New life for an old drug. *Pharmacological Research*.

[14] Sankar G. D., Kondaveni R., Raghava Raju T. V., Vamsi Krishna M. (2009). Gradient stability indicating RP-HPLC method for impurity profiling of simvastatin in tablet dosage forms. *Asian Journal of Chemistry*.

[15] Krishna, Radha S., Deshpande G. R., Rao B. M., Rao N. S. (2010). A Stability-Indicating RP-LC Method for the Determination of Related Substances. *in Simvastatin*.

[16] Plumb R. S., Jones M. D., Rainville P., Castro-Perez J. M. (2007). The rapid detection and identification of the impurities of simvastatin using high resolution sub 2 *μ*m particle LC coupled to hybrid quadrupole time of flight MS operating with alternating high-low collision energy. *Journal of Separation Science*.

[17] Álvarez-Lueje A., Valenzuela C., Squella J. A., Núñez-Vergara L. J. (2005). Stability study of simvastatin under hydrolytic conditions assessed by liquid chromatography. *Journal of AOAC International*.

[18] Vuletić M., Cindrić M., Koružnjak J. D. (2005). Identification of unknown impurities in simvastatin substance and tablets by liquid chromatography/tandem mass spectrometry. *Journal of Pharmaceutical and Biomedical Analysis*.

[19] Ich ICH Topic Q2 (R1) Validation of Analytical Procedures: Text and Methodology.

[20] 9th Pharmacopoeia, European, “Simvastatin monograph No 1563, 01/2017

[21] ICH Stability Testing of New Drug Substances and Products Q1A(R2).

[22] Simões R. G., Diogo H. P., Dias A. (2014). Thermal stability of simvastatin under different atmospheres. *Journal of Pharmaceutical Sciences*.

[23] Rawat T., Pandey I. P. (2015). Forced degradation studies for drug substances and drug products- scientific and regulatory considerations. *Journal of Pharmaceutical Sciences and Research*.

[24] Shinde N. G. (2013). Pharmaceutical Forced Degradation Studies with Regulatory Consideration. *Asian J. Res. Pharm. Sci*.

[25] WHO Technical Report Series

