# Supplementary material for: Comprehensive Assessment of Degradation Behavior of Simvastatin by UHPLC/MS Method, Employing Experimental Design Methodology
Source: Int J Anal Chem. 2018 Aug 8;2018:7170539. doi: 10.1155/2018/7170539 (PMC6106721; doi:10.1155/2018/7170539)
Supplement: Supplementary Materials — As supplementary materials Table S1, Table S2, Table S3, and Table S4 are included. Table S1: structure of the Simvastatin impurities. Table S2: statistical parameters of ANOVA and obtained results from Placket-Burman model. Table S3: the estimates of Box-Behnken regression analysis and statistical parameters of ANOVA. Table S4: Mass spectra fragments of SIM and its impurities. [file 7170539.f1.docx]

| Table S1 Structure of the Simvastatin impurities | | |  |
| --- | --- | --- | --- |
| Compound |  | Structure |  |
| **Simvastatin Impurity A**  IUPAC name  (1S,3R,7S,8S,8aR)-8-{2-[(2R,4R)-4-hydroxy-6-oxooxan-2-yl]ethyl}-3,7-dimethyl-1,2,3,7,8,8a-hexahydronaphthalen-1-yl 2,2-dimethylbutanoate  Formula: C_25_H_38_O_5_  Exact mass: 418.27 Log P: 4.13  Pka: 14.91 |  | 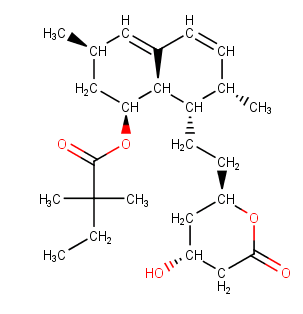 |  |
| **Simvastatin Impurity B**  IUPAC name (1S,3R,7S,8S,8aR)‐8‐{2‐[(2R,4R)‐4‐(acetyloxy)‐6‐oxooxan‐2‐yl]ethyl}‐3,7‐dimethyl‐1,2,3,7,8,8a‐hexahydronaphthalen‐1‐yl 2,2‐dimethylbutanoate  Formula: C_27_H_40_O_6_  Exact mass: 460.61  Log P: 4.56  Pka : / |  | 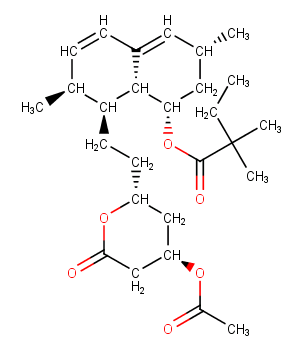 |  |
| **Simvastatin Impurity C**  IUPAC name  (1S,3R,7S,8S,8aR)-3,7-dimethyl-8-{2-[(2R)-6-oxo-3,6-dihydro-2H-pyran-2-yl]ethyl}-1,2,3,7,8,8a-hexahydronaphthalen-1-yl 2,2-dimethylbutanoate  Formula: C_25_H_36_O_4_  Exact mass: 400.26  Log P: 6.18  Pka: / |  | 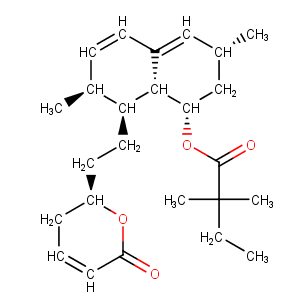 | |
| Compound |  | Structure | |
| **Simvastatin Impurity D**  IUPAC name  (2R,4R)-2-{2-[(2R,6S,8S)-8-[(2,2-dimethylbutanoyl)oxy]-2,6-dimethyl-1,2,6,7,8,8a-hexahydronaphthalen-1-yl]ethyl}-6-oxooxan-4-yl (3R,5R)-7-[(2R,6S,8S)-8-[(2,2-dimethylbutanoyl)oxy]-2,6-dimethyl-1,2,6,7,8,8a-hexahydronaphthalen-1-yl]-3,5 dihydroxyheptanoate  Formula: C_50_H_76_O_10_  Exact mass: 837.15 Log P: 14.73 15.07  Pka: 8.27 |  | 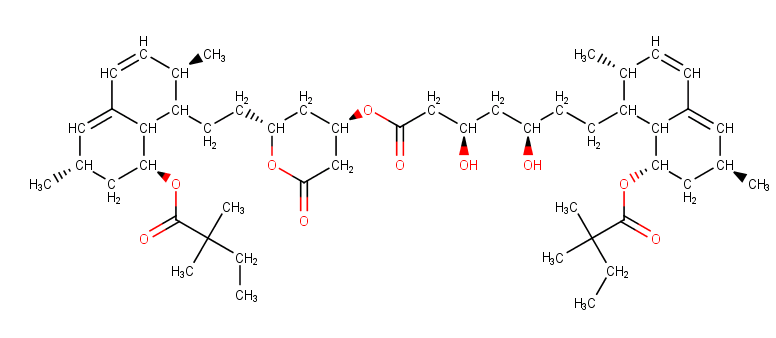 | |
| **Simvastatin Impurity E**  IUPAC name  (1S,3R,7S,8S,8aR)-8-{2-[(2R,4R)-4-hydroxy-6-oxooxan-2-yl]ethyl}-3,7-dimethyl-1,2,3,7,8,8a-hexahydronaphthalen-1-yl (2S)-2-methylbutanoate  Formula: C_24_H_36_O_5_ Exact mass: 404.26 Log P: 3.47  Pka: 14.91 |  | 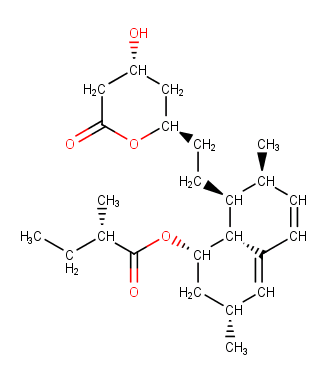 | |
| **Simvastatin Impurity F**  IUPAC name  (1S,3R,7S,8S,8aR)-8-{2-[(2R,4R)-4-hydroxy-6-oxooxan-2-yl]ethyl}-3,7-dimethyl-1,2,3,7,8,8a-hexahydronaphthalen-1-yl (2R)-2-methylbutanoate  Formula: C_24_H_36_O_5_ Exact mass: 404.26 Log P: 3.47  Pka: 14.91 |  | 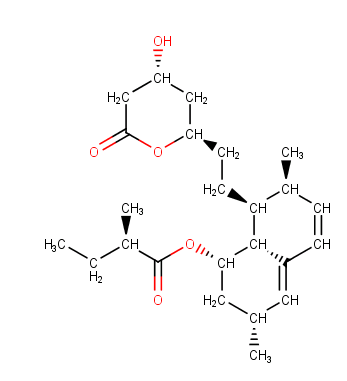 | |
| Compound |  | Structure | |
| **Simvastatin Impurity G**  IUPAC name  (1S,7S,8S,8aR)-8-{2-[(2R,4R)-4-hydroxy-6-oxooxan-2-yl]ethyl}-7-methyl-3-methylidene-1,2,3,7,8,8a-hexahydronaphthalen-1-yl 2,2-dimethylbutanoate  Formula: C_25_H_36_O_5_  Exact mass: 416.26  Log P: 3.74  Pka: 14.91 |  | 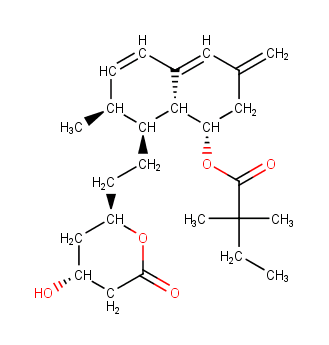 | |
| **Methyl Simvastatin**  IUPAC name  (1S,3R,8S,8aR)-8-{2-[(4R)-4-hydroxy-6-oxooxan-2-yl]ethyl}-1,3,7-trimethyl-1,2,3,7,8,8a-hexahydronaphthalen-1-yl 2,2-dimethylbutanoate  Formula: C_26_H_40_O_5_  Exact mass: 432.29  Log P: 4.21  Pka: 14.91 |  | 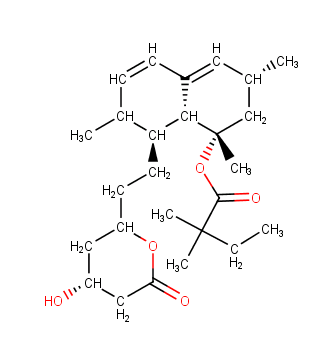 | |
| **Impurity with RRT 1.16**  IUPAC name  ethyl (3R,5R)-7-[(1S,2S,6R,8S,8aR)-8-[(2,2-dimethylbutanoyl)oxy]-2,6-dimethyl-1,2,6,7,8,8a-hexahydronaphthalen-1-yl]-3,5-dihydroxyheptanoate  Formula: C_27_H_44_O_6_  Exact mass: 464.31  Pka: 14.73, 15.47 |  | 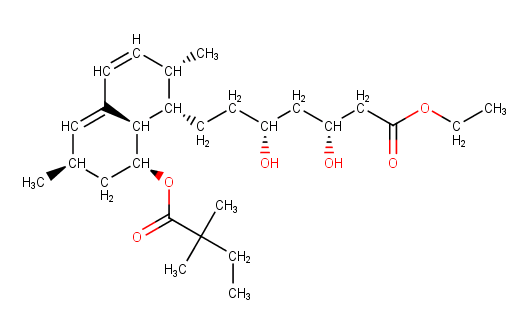 | |

Table S2 Statistical parameters of ANOVA and obtained results from Placket Burman model

| Statistical Parameters | Res E/F | Res G/SIM | Res B/C |
| --- | --- | --- | --- |
| Coefficient of regression (R^2^) | 0.9542 | 0.9533 | 0.9943 |
| Adjusted coefficient of regression | 0.9160 | 0.8972 | 0.9874 |
| Predicted coefficient of regression | 0.7912 | 0.7217 | 0.9589 |
| Predicted residual error sum of squares | 0.20 | 1.48 | 2.21 |
| Adequate Precision | 15.016 | 12.503 | 34.116 |
| Fischer’s ratio (F-ratio) | 24.99 | 17.01 | 144.62 |
| P value | 0.0006 | 0.0035 | 0.0001 |
| Coefficient of variation | 8.00 | 6.47 | 8.18 |

Table S3 The estimates of Box-Behnken regression analysis and statistical parameters of ANOVA

| Statistical Parameters | | | | | | Rs G/SIM  (*y_1_*) | | | T  (*y_2_*) | | | Rs B/C  (*y_3_*) | |
| --- | --- | --- | --- | --- | --- | --- | --- | --- | --- | --- | --- | --- | --- |
| Coefficient of regression (R^2^) | | | | | | 0.9281 | | | 0.9765 | | | 0.9258 | |
| Adjusted coefficient of regression | | | | | | 0.8741 | | | 0.9453 | | | 0.8846 | |
| Predicted coefficient of regression | | | | | | 0.7753 | | | 0.8434 | | | 0.7689 | |
| Lack of fit | | | | | | 0.3095 | | | 0.6747 | | | 0.0176 | |
| Predicted residual error sum of squares | | | | | | 1.26 | | | 0.065 | | | 0.24 | |
| Adequate Precision | | | | | | 13.772 | | | 15.952 | | | 12.902 | |
| Fischer’s ratio (F-ratio) | | | | | | 17.20 | | | 31.23 | | | 22.45 | |
| P value | | | | | | 0.0004 | | | < 0.0001 | | | <0.0001 | |
| Coefficient of variation | | | | | | 6.17 | | | 3.15 | | | 5.67 | |
| Regression coefficient | | | | | | | | | | | | | |
|  | *b*_0_ | | *b*_1_ | | *b*_2_ | *b*_3_ | *b*_12_ | *b*_13_ | *b*_23_ | *b*_11_ | *b*_22_ | | *b*_33_ |
| RsG/SIM(*y_1_*) | 4.45 | | / | | 0.29 | -0.25 | / | -0.61 | / | -0.52 | -0.55 | | -0.46 |
| T (*y_2_*) | | 1.07 | | -0.074 | -0.042 | 0.051 | -0.11 | 0.15 | / | 0.094 | 0.096 | | 0.20 |
| Rs B/C (*y_3_*) | | 2.11 | | -0.076 | / | / | / | / | 0.10 | -0.35 | -0.20 | | -0.33 |

Table S4 Mass spectra fragments of SIM and its impurities

| Name of impurity | Rt (min) | RRt | λ_max_ (nm) | MW | MS | MS^2^ | MS^3^ |
| --- | --- | --- | --- | --- | --- | --- | --- |
| Simvastatin Impurity A | 9.20 | 0.67 | 235 | 458.56 | **459** | 319; **419;** | 199; 267; 285; **302** |
| Simvastatin Impurity E | 12.36 | 0.89 | 236 | 404.54 | **406.43** | **267;** 285; | **199;** 303; |
| Simvastatin Impurity F | 12.60 | 0.90 | 236 | 404.54 | **417** | **267;** 285; | **199;** 303; |
| Simvastatin Impurity G | 13.31 | 0.95 | 234 | 416.52 | 416,**434** | **199;** 285; 303; | **143;** 157; |
| Simvastatin | 14.01 | 1.00 | 238 | 418.57 | 419;**436**;855 | 319; **419**; | 199; 267; 285; **302**; |
| Methyl Simvastatin | 15.07 | 1.08 | 238 | 432.60 | 433;**451** | 285; 335; **433**; | 199; 299; **335**; |
| Impurity RRT 1.16 | 15.72 | 1.16 | 238 | 465 | 419;438; **465; 349** | **313**; 331; 446; | **249**; 267; 295; |
| Simvastatin Impurity B | 16.86 | 1.20 | 234 | 460.60 | 418;**460** | 285; **372**; 418; | 133; **199**; 285; |
| Simvastatin Impurity C | 17.38 | 1.24 | 234 | 400.56 | **436** | **285**; 303; | 199; **285**; |
| Simvastatin Impurity D | 23.86 | 1.70 | 238 | 837.16 | **419;** 855; | 319; **419**; | 199; 267; 285; **302**; |
| Most abundant ions in mass spectra are market with bold. Additional 17 amu are detected as adduct of NH_3_^+^ from mobile phase | | | | | | | |
